# Association between cardiopulmonary exercise capacity and clinical parameters in post-PCI patients with coronary artery disease from Fujian, China

**DOI:** 10.3389/fcvm.2025.1674787

**Published:** 2025-09-25

**Authors:** LiHan Lin, Delong Li, YiPing Liu, GuoPeng Hu, Wei Zheng, ZuLin Chen, YiKun Zheng, YongDa Dong

**Affiliations:** ^1^ Provincial University Key Laboratory of Sport and Health Science, School of Physical Education and Sport Science, Fujian Normal University, Fuzhou, China; ^2^ College of Physical Education, Huaqiao University, Quanzhou, China; ^3^ Department of Cardiology, Quanzhou First Hospital Affiliated to Fujian Medical University, Quanzhou, China; ^4^ School of Physical Education and Health Care, Sanming University, Sanming, China

**Keywords:** cardiopulmonary exercise testing, peak oxygen uptake, coronary artery disease, percutaneous coronary intervention, predictors, cardiac rehabilitation, linear regression

## Abstract

**Background:**

Peak oxygen uptake (VO₂peak) assessed by cardiopulmonary exercise testing (CPET) is a key indicator of functional capacity and prognosis in patients with coronary artery disease (CAD) following percutaneous coronary intervention (PCI). However, the clinical predictors of exercise capacity among post-PCI patients in Fujian, China, remain insufficiently characterized. Identifying such predictors can enhance individualized rehabilitation strategies and secondary prevention measures in clinical practice.

**Methods:**

This retrospective study analyzed 575 CAD patients who underwent PCI and completed CPET within six weeks post-procedure at Quanzhou First Hospital Affiliated to Fujian Medical University from June 2020 to June 2024. Participants' demographics, medical history, echocardiographic parameters, and laboratory results were collected. Univariable and multivariable linear regression identified independent predictors of VO₂peak, with subgroup analyses by age (<65 vs. ≥65 years) and gender.

**Results:**

The mean VO₂peak of the study population was 19.29 ± 4.41 ml/kg/min. Independent predictors of lower VO₂peak included older age (*β* = –0.06, *P* < 0.001), female sex (*β* = –1.71, *P* < 0.001), acute coronary syndrome (ACS; *β* = –1.01, *P* < 0.001), smoking (*β* = –2.37, *P* < 0.001), hypertension (*β* = –0.82, *P* = 0.004), higher resting heart rate (RHR; *β* = –0.10, *P* < 0.001), and lower hematocrit (HCT; *β* = –0.20, *P* = 0.002). Conversely, higher red blood cell (RBC) count (*β* = 1.20, *P* = 0.012) and hemoglobin (Hb; *β* = 0.09, *P* < 0.001) levels predicted better exercise capacity. Subgroup analyses highlighted age- and sex-specific determinants: notably, lower main pulmonary artery diameter (MPA) and lower HCT uniquely affected younger patients, while hypertension primarily impacted older patients. Gender-specific associations revealed that hypertension and high-density lipoprotein cholesterol (HDL-C) predicted VO₂peak in males, whereas lower body weight, higher RBC, and lower HCT were significant in females.

**Conclusion:**

Significant demographic, clinical, echocardiographic, and biochemical predictors of cardiopulmonary exercise capacity were identified among post-PCI CAD patients from Fujian, China. Age- and sex-specific differences underline the necessity for personalized rehabilitation and prevention strategies to improve cardiopulmonary fitness and clinical outcomes in this population.

## 1 Introduction

Coronary artery disease (CAD) remains a leading cause of morbidity and mortality worldwide, particularly in China, where its prevalence continues to rise alongside an aging population (1, 2). Percutaneous coronary intervention (PCI) has markedly improved clinical outcomes by rapidly restoring myocardial perfusion and reducing infarct size (3). Nevertheless, recent studies indicate that more than half of post-PCI patients experience a clinically meaningful decline in cardiorespiratory fitness and exercise capacity after revascularization (4–6)—an issue often overlooked in routine clinical practice, underscoring the need to identify factors that influence postoperative functional recovery.

Cardiopulmonary exercise testing (CPET), which integrates cardiovascular, pulmonary, and musculoskeletal responses, provides an objective assessment of functional capacity (7, 8). Its principal metric, peak oxygen uptake (VO₂peak), is not only a robust prognostic marker for cardiovascular disease but also a cornerstone for tailoring exercise prescriptions and predicting major adverse cardiovascular events in cardiac rehabilitation programmes (9–11). Cardiopulmonary exercise capacity is thus essential for tracking recovery in post-PCI CAD patients. However, the clinical implementation of CPET is often constrained by its high cost, technical complexity, and limited patient compliance. These challenges are especially pronounced in China and other developing countries, where large populations and disparities in healthcare resources further hinder widespread use. Therefore, identifying easily accessible clinical predictors of exercise capacity may help guide risk assessment and rehabilitation strategies, particularly for patients unable to undergo CPET. Prior studies have identified several determinants of cardiopulmonary exercise capacity, including age, sex, body mass index (BMI), and the presence of chronic conditions (12–15). However, few studies have specifically investigated factors influencing cardiopulmonary exercise capacity in post-PCI CAD patients, particularly within the population of Fujian Province, China.

Building on these observations, we hypothesized that, in post-PCI CAD patients, measures of cardiac function and blood biochemistry would independently predict VO₂peak, in addition to demographic and medical history variables, and that these associations would differ by sex and age. To test this hypothesis, we assessed VO₂peak and its predictors in post-PCI CAD patients from Fujian, China. Clarifying these determinants will inform targeted rehabilitation strategies and reinforce secondary prevention after PCI.

## 2 Methods

### 2.1 Data source and participants

This study retrospectively analyzed data from 1,253 patients with CAD who underwent PCI (“post-PCI patients”) during hospitalization at Quanzhou First Hospital Affiliated to Fujian Medical University between June 2020 and June 2024. The diagnosis of CAD was established according to the American Heart Association (AHA) criteria (16, 17), defined as a stenosis of ≥50% in any major coronary artery (left anterior descending, left circumflex, or right coronary artery) or its main branches. Both the diagnosis of CAD and the performance of PCI were verified by the research team through review of the hospital information system records.

**Figure 1:**
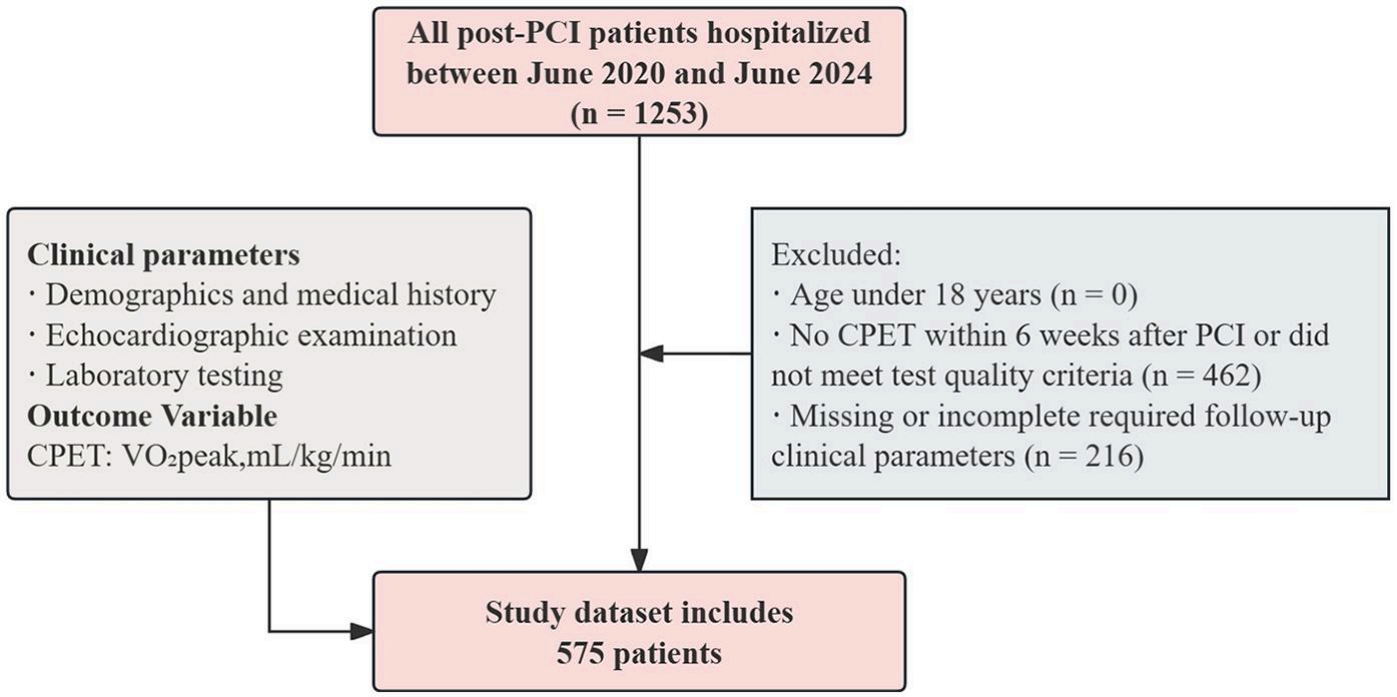
The flow chart for study population selection.

For study purposes, participants were eligible if they met all of the following criteria: (1) Age >18 years; (2) Underwent CPET within 6 weeks after PCI and met the predefined test quality criteria, defined as a symptom-limited test with a respiratory exchange ratio (RER) >1.05 and/or achievement of 75%–85% of the age-predicted maximal heart rate under continuous monitoring; (3) Completed transthoracic echocardiography, complete blood count, and routine biochemical assessments at follow-up. Exclusion criteria were: (1) Incomplete follow-up clinical parameters; (2) Severe cardiac or systemic comorbidities (including but not limited to recurrent angina or chest pain after PCI, cardiogenic shock, severe arrhythmia, active endocarditis, myocarditis, pericarditis, severe aortic stenosis, malignancy, severe anemia, acute infection, advanced hepatic or renal dysfunction, moderate-to-severe chronic lung disease, pulmonary embolism, or interstitial lung disease); (3) use of cardiac assist devices (such as intra-aortic balloon pump or left ventricular assist devices) during hospitalization; (4) inability or refusal to perform CPET (e.g., severe musculoskeletal disorders, significant physical disability, or severe psychiatric or cognitive disorders). A total of 575 post-PCI patients were included in the final analysis. The detailed selection flow is illustrated in Figure 1.

This study was conducted in accordance with the principles of the Declaration of Helsinki and approved by the Ethics Committee of Quanzhou First Hospital Affiliated to Fujian Medical University [Approval No. K052(2023)]. As a retrospective study, the requirement for informed consent was waived.

### 2.2 Measurements

#### 2.2.1 Cardiopulmonary exercise capacity

Cardiopulmonary exercise capacity was assessed by measuring VO₂peak, which is consistent with previous studies (18–20). In this study, the VO₂peak was determined using a maximal symptom-limited incremental CPET performed on a cycle ergometer with the Vyntus CPX system (Vyaire Medical GmbH, Leibnizstrasse 7, 97204 Hoechberg, Germany), which was defined as the highest consumption of O2, obtained by averaging data from the last 30 s of peak effort.

A strictly graded ramp protocol was applied, and the equipment was calibrated daily before clinical examinations (21–23). The protocol began with a 3 min rest period, followed by a 3 min warm-up at 0 W. Thereafter, the workload was increased continuously at a rate of 10–30 W/min, individualized by the supervising physician according to the participant's health status, physical fitness, and anticipated exercise tolerance until volitional exhaustion. Participants were instructed to maintain a pedaling cadence of 55–65 revolutions per minute. The total duration of the test was typically 10–15 min. Blood pressure, electrocardiogram (ECG), and respiratory gas exchange parameters were continuously monitored and recorded throughout the test. All assessments were supervised by an experienced cardiologist. The test was terminated upon achievement of 75%–85% of the age-predicted maximal heart rate or when the RER exceeded 1.05. If the target heart rate was not reached or the RER remained ≤1.05, the test was stopped under any of the following conditions: (1) The occurrence of symptoms such as chest tightness, chest pain, dyspnea, dizziness, lower-limb pain, or fatigue precluding further exercise; (2) Horizontal or downsloping ST-segment depression ≥0.20 mV in contiguous leads lasting >1 min, or ST-segment elevation ≥0.1 mV with an arched upward configuration; (3) A decrease in systolic blood pressure >10 mmHg during exercise; (4) Excessive elevation in blood pressure, defined as systolic blood pressure >200 mmHg or diastolic blood pressure >110 mmHg; (5) The development of ventricular tachycardia, supraventricular tachycardia, frequent ventricular premature beats, second- or third-degree atrioventricular block, or significant sinus bradycardia. Additional protocol details are provided in the **Methods supplement (**Supplementary Material S2) to ensure reproducibility.

#### 2.2.2 Demographics and medical history

A total of 12 demographic and clinical characteristics were collected: age, height, weight, gender, type of CAD, smoking status, comorbidities (hypertension, hyperlipidemia, diabetes), BMI, resting heart rate (RHR), and CPET timing.

CAD was classified as stable CAD or acute coronary syndrome (ACS), based on the primary diagnosis in medical records, consistent with previous studies (24, 25). Smoking was defined per the WHO criteria: individuals with continuous or cumulative smoking for over six months were considered smokers (22). Hypertension was defined as either: (1) systolic BP ≥140 mmHg and/or diastolic BP ≥90 mmHg without antihypertensive therapy (26); or (2) self-reported physician diagnosis. Hyperlipidemia was defined as: (1) TC ≥6.22 mmol/L, LDL-C ≥ 4.14 mmol/L, TG ≥2.26 mmol/L, or HDL-C below threshold (men: <1.04 mmol/L; women: <1.30 mmol/L); or (2) self-reported diagnosis (27). Diabetes was defined as: (1) FPG ≥7.0 mmol/L or HbA1c ≥ 6.5%; (2) positive oral glucose tolerance test (OGTT); or (3) self-reported diagnosis (28). BMI is classified as normal (18.5–22.9 kg/m²), overweight (23.0–24.9 kg/m²), and obese (≥25.0 kg/m²), according to WHO recommendations for Asian populations (29). RHR was measured by 24 h resting ECG monitoring closest to the CPET date after PCI, using the PageWriter TC70 system (Philips, Amsterdam, Netherlands) (30). CPET timing was categorized as <1 week, 1–3 weeks, and 4–6 weeks after PCI.

#### 2.2.3 Echocardiographic examination

Transthoracic 2-dimensional, M-mode, and Doppler echocardiographic examinations were performed after PCI using digital ultrasound systems (EPIQ5C/EPIQ7C, Philips, Amsterdam, Netherlands; Vivid-7, GE Healthcare, Chicago, IL) equipped with a phased array transducer. A total of 24 parameters were assessed, including left ventricular end-diastolic diameter (LVEDD), left ventricular end-systolic diameter (LVESD), interventricular septal diastolic thickness (IVSd), left ventricular posterior wall diastolic thickness (LVPWd), left atrial anteroposterior diameter (LA-ap), aortic annulus diameter (AO-a), aortic sinus diameter (AO-s), proximal ascending aorta diameter (AO-asc), main pulmonary artery diameter (MPA), left ventricular end-diastolic volume (EDV), left ventricular end-systolic volume (ESV), ejection fraction (EF), fractional shortening (FS), stroke volume (SV), cardiac output (CO), cardiac index (CI), early mitral inflow velocity (MV E), late mitral inflow velocity (MV A), E/A ratio, aortic valve flow velocity (AV), pulmonary artery flow velocity (PV), early diastolic septal mitral annulus velocity (e′s), early diastolic lateral mitral annulus velocity (e′l), and E/e′ ratio. These echocardiographic indices provide a comprehensive assessment of cardiac structure and function (31).

#### 2.2.4 Laboratory testing

All laboratory measurements were performed at the central laboratory of the First Hospital of Quanzhou. Complete blood count parameters, including red blood cell (RBC) count, hemoglobin (Hb), hematocrit (HCT), and mean corpuscular volume (MCV), were measured using automated analyzers (XW-100, Sysmex, Kobe, Japan; DxH 560AL, Beckman Coulter, Brea, CA, USA). Serum triglycerides (TG), total cholesterol (TC), low-density lipoprotein cholesterol (LDL-C), high-density lipoprotein cholesterol (HDL-C), fasting plasma glucose (FPG), and creatine kinase (CK) were determined using automated biochemical analyzers (LABOSPECT-006/7180, Hitachi, Tokyo, Japan). For each patient, the results were obtained from the laboratory testing performed closest to the CPET following PCI.

### 2.3 Statistical analysis

Data analyses were conducted using SPSS (version 26.0; IBM Corp., Armonk, NY, USA). Continuous variables were tested for normality using the Shapiro–Wilk test and expressed as medians (IQR) due to non-normal distributions. Categorical variables were presented as frequencies (%). Baseline characteristics across VO₂peak quartiles were compared using Kruskal–Wallis and Chi-square tests. VO₂peak was divided into quartiles based on sample distribution (25th, 50th, and 75th percentiles), since no universally accepted clinical cutoffs exist in post-PCI patients. Univariable linear regression was used to assess associations of clinical, echocardiographic, and laboratory parameters with VO₂peak. Variables with *P* < 0.05 were subsequently entered into a multivariable linear regression model using backward stepwise selection to identify independent predictors, reported as β-coefficients with 95% CI. Multicollinearity was evaluated using variance inflation factors (VIF), with all retained predictors showing VIF < 5. Regression assumptions, including linearity, homoscedasticity, and normality of residuals, were verified both graphically and through statistical tests. Subgroup analyses were conducted according to age [<65 years and ≥65 years, given the sample's median age of 64.0 (56.0–70.0) years and consistent with cardiovascular guidance and prior PCI/CAD studies (32–35)] and gender (male and female), using multivariable regression models. Sensitivity analyses compared the demographics and medical history of included and excluded patients. Statistical significance was set at *P* < 0.05 (two-tailed).

## 3 Results

### 3.1 Characteristics of the study population

According to the inclusion and exclusion criteria, 575 participants [female: 32.52%; median age: 64.0 years (IQR: 56.0–70.0); the median interval from the last PCI to CPET was 18 days (IQR: 9–27 days); mean VO₂peak: 19.29 ± 4.41 ml/kg/min, males: 20.21 ± 4.54 ml/kg/min, females: 17.37 ± 3.41 ml/kg/min] were included in this study. Participants were stratified into quartiles based on VO₂peak. Those in the lowest quartile were older, shorter in height, had higher RHR, and lower Hb and HCT compared with higher quartiles (all *P* < 0.001). The prevalence of hypertension, diabetes, and ACS was higher in the lowest quartile, whereas the proportion of males and stable CAD increased across higher quartiles. Significant differences were also observed in multiple echocardiographic and laboratory parameters among groups. Detailed characteristics are summarized in Table 1. The results of the Shapiro–Wilk normality tests for all continuous variables are shown in Supplementary Table S1, indicating that none of the variables were normally distributed (all *P* < 0.05). Descriptive statistics for key CPET-derived variables are summarized in Supplementary Table S2.

**Table 1:** The characteristics of study participants.

Characteristics	Peak Oxygen Uptake, ml/kg/min						
	Total (*n* = 575)	Q1 (9.90–16.30, *n* = 149)	Q2 (16.30–18.90, *n* = 143)	Q3 (18.90–21.75, *n* = 139)	Q4 (21.75–36.60, *n* = 144)	Statistic	*P*
Demographics and medical history							
Age, years	64.00 (56.00, 70.00)	70.00 (61.00,75.00)	65.00 (57.50,71.00)	63.00 (56.00,68.50)	59.00 (53.00,65.25)	H = 59.69	**<** **.** **001**
Hight, cm	165.00 (160.00, 170.00)	163.00 (157.00,169.00)	165.00 (160.00,170.00)	165.00 (160.00,170.00)	168.50 (165.00,172.25)	H = 41.41	**<** **.** **001**
Weight, kg	67.00 (60.00, 75.00)	67.00 (60.00,75.00)	67.00 (59.50,75.00)	67.00 (60.00,77.00)	67.00 (60.00,76.00)	H = 0.34	0.953
Gende						*χ*² = 54.43	**<** **.** **001**
Male	388 (67.48)	75 (50.34)	86 (60.14)	99 (71.22)	128 (88.89)		
Female	187 (32.52)	74 (49.66)	57 (39.86)	40 (28.78)	16 (11.11)		
Type of CAD						χ² = 18.24	**<** **.** **001**
SA	354 (61.57)	78 (52.35)	79 (55.24)	90 (64.75)	107 (74.31)		
ACS	221 (38.43)	71 (47.65)	64 (44.76)	49 (35.25)	37 (25.69)		
Smoking						χ² = 28.11	**<** **.** **001**
No	394 (68.52)	79 (53.02)	95 (66.43)	107 (76.98)	113 (78.47)		
Yes	181 (31.48)	70 (46.98)	48 (33.57)	32 (23.02)	31 (21.53)		
Hypertension						χ² = 45.26	**<** **.** **001**
No	267 (46.43)	49 (32.89)	47 (32.87)	79 (56.83)	92 (63.89)		
Yes	308 (53.57)	100 (67.11)	96 (67.13)	60 (43.17)	52 (36.11)		
Hyperlipidemia						χ² = 2.87	0.413
No	223 (38.78)	50 (33.56)	56 (39.16)	55 (39.57)	62 (43.06)		
Yes	352 (61.22)	99 (66.44)	87 (60.84)	84 (60.43)	82 (56.94)		
Diabetes						χ² = 11.70	**0** **.** **008**
No	435 (75.65)	104 (69.80)	102 (71.33)	106 (76.26)	123 (85.42)		
Yes	140 (24.35)	45 (30.20)	41 (28.67)	33 (23.74)	21 (14.58)		
CPET timing						χ² = 8.92	0.178
<1 week	83 (14.43)	19 (12.75)	24 (16.78)	20 (14.39)	20 (13.89)		
1–3 weeks	318 (55.30)	79 (53.02)	72 (50.35)	74 (53.24)	93 (64.58)		
4–6 weeks	174 (30.26)	51 (34.23)	47 (32.87)	45 (32.37)	31 (21.53)		
BMI						-	**0** **.** **016**
Underweight	17 (2.96)	6 (4.03)	4 (2.80)	2 (1.44)	5 (3.47)		
Normal weight	221 (38.43)	39 (26.17)	56 (39.16)	56 (40.29)	70 (48.61)		
Overweight	235 (40.87)	73 (48.99)	57 (39.86)	53 (38.13)	52 (36.11)		
Obesity	102 (17.74)	31 (20.81)	26 (18.18)	28 (20.14)	17 (11.81)		
Echocardiographic examination							
LVEDD, mm	47.00 (45.00, 50.00)	46.00 (44.00,49.00)	47.00 (44.00,50.00)	47.00 (45.00,50.00)	48.00 (45.00,50.25)	H = 4.73	0.193
LVESD, mm	29.00 (27.00, 32.00)	29.00 (27.00,31.00)	29.00 (27.00,32.00)	30.00 (27.00,32.00)	30.00 (28.00,32.00)	H = 3.06	0.383
IVSd, mm	9.00 (9.00, 10.00)	9.00 (9.00,10.00)	10.00 (9.00,10.00)	9.00 (8.50,10.75)	9.00 (8.00,10.00)	H = 2.64	0.451
LVPWd, mm	9.00 (8.00, 10.00)	9.00 (9.00,10.00)	9.00 (9.00,10.00)	9.00 (8.00,10.00)	9.00 (8.00,10.00)	H = 0.53	0.912
LA-ap, mm	34.00 (31.00, 37.00)	35.00 (31.00,38.00)	34.00 (31.00,37.00)	34.00 (31.00,37.00)	34.00 (31.00,37.00)	H = 1.70	0.636
AO-a, mm	21.00 (19.00, 22.00)	20.00 (19.00,21.00)	20.00 (19.00,21.00)	21.00 (19.00,22.00)	21.00 (20.00,22.00)	H = 7.20	0.066
AO-s, mm	33.00 (31.00, 36.00)	33.00 (31.00,36.00)	34.00 (31.00,36.00)	33.00 (30.50,35.00)	33.00 (31.00,36.00)	H = 2.35	0.502
AO-asc, mm	33.00 (30.00, 35.00)	33.00 (31.00,36.00)	33.00 (30.00,35.00)	32.00 (30.00,35.00)	33.00 (30.00,35.00)	H = 4.30	0.231
MPA, mm	22.00 (21.00, 24.00)	22.00 (21.00,24.00)	22.00 (21.00,23.40)	22.00 (21.00,23.00)	22.00 (21.00,23.00)	H = 5.19	0.158
EDV, ml	102.00 (92.00, 118.00)	97.00 (88.00,113.00)	102.00 (88.00,118.00)	102.00 (92.00,118.00)	108.00 (92.00,119.50)	H = 5.21	0.157
ESV, ml	34.00 (28.00, 41.00)	32.00 (27.00,41.00)	32.00 (27.00,41.00)	35.00 (28.00,41.00)	35.00 (29.00,42.25)	H = 3.17	0.366
EF, %	67.00 (62.00, 71.00)	66.00 (62.00,71.00)	68.00 (63.00,72.00)	67.00 (62.00,72.00)	66.00 (62.00,71.00)	H = 2.73	0.435
FS, %	38.00 (34.00, 40.00)	37.00 (34.00,40.00)	38.00 (35.00,41.00)	38.00 (33.00,41.00)	37.00 (33.00,40.00)	H = 2.82	0.42
SV, ml	67.00 (59.00, 77.00)	66.00 (57.00,74.00)	67.00 (59.00,77.50)	68.00 (58.50,78.00)	70.00 (60.00,77.00)	H = 4.73	0.193
CO, ml/min	4,830.00 (4,094.50, 5,643.00)	4,793.40 (4,116.00,5,520.00)	4,872.00 (4,058.50,5,710.80)	4,913.60 (4,058.00,5,625.50)	4,817.00 (4,258.00,5,705.00)	H = 0.32	0.956
CI, ml/min/m²	2,833.00 (2,432.00, 3,226.50)	2,834.80 (2,417.00,3,265.00)	2,846.80 (2,452.00,3,274.70)	2,850.00 (2,426.50,3,253.50)	2,781.00 (2,411.50,3,187.25)	H = 0.61	0.895
MV E, m/s	0.70 (0.60, 0.80)	0.70 (0.60,0.80)	0.70 (0.60,0.80)	0.70 (0.60,0.80)	0.70 (0.60,0.80)	H = 4.53	0.21
MV A, m/s	0.80 (0.70, 0.90)	0.90 (0.70,1.00)	0.80 (0.70,0.90)	0.80 (0.68,0.90)	0.70 (0.60,0.80)	H = 42.78	**<** **.** **001**
E/A	0.86 (0.71, 1.00)	0.78 (0.64,1.00)	0.83 (0.71,1.00)	0.86 (0.71,1.06)	0.88 (0.73,1.14)	H = 15.16	**0** **.** **002**
AV, m/s	1.20 (1.10, 1.40)	1.20 (1.10,1.40)	1.30 (1.10,1.40)	1.20 (1.10,1.40)	1.20 (1.10,1.30)	H = 8.79	**0** **.** **032**
PV, m/s	0.90 (0.80, 1.00)	0.90 (0.80,1.00)	0.90 (0.80,1.10)	0.90 (0.80,1.00)	0.90 (0.80,1.00)	H = 8.10	**0** **.** **044**
e's, cm/s	7.00 (5.00, 8.00)	6.00 (5.00,7.00)	6.00 (5.00,8.00)	7.00 (6.00,7.00)	7.00 (6.00,8.00)	H = 22.93	**<** **.** **001**
e'l, cm/s	9.00 (7.00, 11.00)	8.00 (7.00,10.00)	9.00 (7.00,10.50)	9.00 (7.50,11.00)	10.00 (8.00,11.00)	H = 16.04	**0** **.** **001**
E/e	9.00 (7.00, 11.00)	9.00 (8.00,11.00)	9.00 (8.00,11.00)	9.00 (7.00,11.00)	8.00 (7.00,9.00)	H = 25.21	**<** **.** **001**
Laboratory testing							
RHR, bpm	74.00 (69.00, 82.00)	81.00 (74.00,90.00)	83.00 (73.50,92.00)	71.00 (67.00,74.00)	71.00 (68.00,74.00)	H = 151.75	**<** **.** **001**
RBC, 10^12^/L	4.66 (4.30, 4.97)	4.40 (4.08,4.76)	4.57 (4.26,4.87)	4.75 (4.46,4.99)	4.85 (4.60,5.15)	H = 69.45	**<** **.** **001**
Hb, g/L	141.00 (131.50, 151.00)	132.00 (123.00,142.00)	137.00 (129.00,145.00)	144.00 (136.50,152.00)	149.00 (142.00,158.00)	H = 110.82	**<** **.** **001**
HCT, %	41.40 (38.40, 44.00)	39.30 (36.50,42.50)	40.70 (37.80,42.55)	41.60 (39.75,44.40)	43.60 (41.22,45.52)	H = 76.42	**<** **.** **001**
MCV, fL	88.70 (86.40, 91.60)	88.80 (86.50,92.30)	88.60 (86.35,90.55)	88.50 (85.90,91.65)	89.25 (86.57,92.23)	H = 4.62	0.202
TG, mmol/L	1.34 (0.98, 2.09)	1.43 (1.08,2.00)	1.31 (0.90,2.04)	1.36 (0.95,2.26)	1.25 (0.94,2.01)	H = 1.50	0.683
TC, mmol/L	4.77 (3.75, 5.83)	4.65 (3.59,5.89)	4.48 (3.57,5.48)	5.04 (4.03,5.92)	4.87 (3.97,6.06)	H = 8.46	**0** **.** **037**
LDL-C, mmol/L	2.91 (2.12, 3.73)	2.85 (1.89,3.73)	2.83 (2.09,3.57)	3.23 (2.41,3.86)	2.92 (2.25,3.79)	H = 7.07	0.07
HDL-C, mmol/L	1.29 (1.10, 1.58)	1.27 (1.09,1.58)	1.27 (1.10,1.54)	1.27 (1.10,1.52)	1.35 (1.16,1.62)	H = 5.16	0.161
FBG, mmol/L	5.59 (5.01, 6.87)	5.54 (4.98,7.63)	5.66 (5.12,7.29)	5.70 (5.17,6.79)	5.38 (4.90,6.01)	H = 9.27	**0** **.** **026**
CK, U/L	94.00 (66.00, 129.50)	88.00 (62.00,112.00)	89.00 (63.00,129.00)	97.00 (69.00,126.50)	109.50 (75.00,148.25)	H = 11.88	**0** **.** **008**

Values are expressed as median (IQR). VO₂peak quartiles were defined as Q1 (≤16.30), Q2 (16.30–18.90), Q3 (18.90–21.75), and Q4 (≥21.75 ml·kg⁻¹·min⁻¹) based on sample distribution. Continuous variables are shown as median (IQR); categorical variables as *n* (%). H: Kruskal-waills test, χ²: Chi-square test; -: Fisher exact test. Reference ranges: LVEDD 42–58 mm (men), 38–52 mm (women); LVESD 25–40 mm (men), 22–35 mm (women); IVSd 6–10 mm (men), 6–9 mm (women); LVPWd 6–10 mm (men), 6–9 mm (women); LA-ap ≤40 mm; aortic sinus 29–39 mm (men), 27–35 mm (women); ascending aorta 26–36 mm (men), 23–33 mm (women); EDV 67–155 ml (men), 46–106 ml (women); ESV 22–58 ml (men), 14–42 ml (women); EF 55%–70%; FS 28%–44%; SV 60–100 ml; CO 4–8 L/min; CI 2.5–4.0 L/min/m²; E/A ≈ 1.0–2.0 (age dependent); AV ≤2.0 m/s; PV 0.6–0.9 m/s; e′s ≥ 7 cm/s; e′l ≥ 10 cm/s; E/e < 14; RHR 60–100 bpm; RBC 4.2–5.9 × 10¹²/L (men), 3.9–5.0 × 10¹²/L (women); Hb 130–170 g/L (men), 120–150 g/L (women); HCT 40%–50% (men), 36%–44% (women); MCV 80–100 fL; TG <1.7 mmol/L; TC <5.2 mmol/L; LDL-C < 3.4 mmol/L; HDL-C ≥ 1.0 mmol/L (men), ≥1.3 mmol/L (women); FBG 3.9–6.1 mmol/L; CK 40–200 U/L (men), 26–190 U/L (women, lab dependent).

*P* Values in bold indicate statistical significance at *P* < 0.05.

### 3.2 Univariable linear regression analysis

Univariable linear regression identified 20 variables significantly associated with VO₂peak as a continuous outcome (Table 2). In the category of demographic information, VO₂peak was significantly lower in individuals with higher age (*β* = –0.15, *P* < 0.001), height (*β* = 0.15, *P* < 0.001), female sex (*β* = –2.83, *P* < 0.001), ACS (*β* = –1.53, *P* < 0.001), smoking (*β* = –2.06, *P* < 0.001), hypertension (*β* = –2.14, *P* < 0.001), diabetes (*β* = –1.30, *P* = 0.002), and elevated RHR (*β* = –0.14, *P* < 0.001). For echocardiographic examination, VO₂peak was positively associated with LVEDD (*β* = 0.09, *P* = 0.039), EDV (*β* = 0.02, *P* = 0.031), E/A (*β* = 2.24, *P* < 0.001), e′ₛ (*β* = 0.40, *P* < 0.001), and e′ₗ (*β* = 0.28, *P* < 0.001), while E/e′ (*β* = –0.30, *P* < 0.001) and MV A (*β* = –6.48, *P* < 0.001) were negatively associated. In terms of laboratory testing, higher levels of RBC (*β* = 3.30, *P* < 0.001), Hb (*β* = 0.13, *P* < 0.001), HCT (*β* = 0.32, *P* < 0.001), HDL-C (*β* = 0.05, *P* = 0.021), and CK (*β* = 0.01, *P* = 0.029) were positively associated with VO₂peak.

**Table 2:** Univariable linear regression of clinical parameters associated with peak VO₂.

Characteristics	S.E	t	P	*β* (95% CI)
Demographics and medical history				
Age, years	0.02	−8.46	<.001	−0.15 (−0.18∼−0.11)
Hight, cm	0.02	6.24	<.001	0.15 (0.10∼0.19)
Weight, kg	0.02	0.55	0.584	0.01 (−0.02∼0.04)
Gende				
Male				0.00 (Reference)
Female	0.37	−7.56	<.001	−2.83 (−3.56∼−2.10)
Type of CAD				
SA				0.00 (Reference)
ACS	0.37	−4.09	<.001	−1.53 (−2.26∼−0.79)
Smoking				
No				0.00 (Reference)
Yes	0.39	−5.32	<.001	−2.06 (−2.82∼−1.30)
Hypertension				
No				0.00 (Reference)
Yes	0.36	−5.98	<.001	−2.14 (−2.84∼−1.44)
Hyperlipidemia				
No				0.00 (Reference)
Yes	0.38	−1.11	0.267	−0.42 (−1.16∼0.32)
Diabetes				
No				0.00 (Reference)
Yes	0.42	−3.07	0.002	−1.30 (−2.14∼−0.47)
CPET timing				
<1 week				0.00 (Reference)
1–3 weeks	0.54	0.27	0.790	0.14 (−0.92∼1.21)
4–6 weeks	0.59	−0.89	0.375	−0.52 (−1.67∼0.63)
BMI				
Underweight				0.00 (Reference)
Normal weight	1.09	1.53	0.128	1.67 (−0.48∼3.81)
Overweight	1.09	0.12	0.903	0.13 (−2.01∼2.27)
Obesity	1.14	−0.11	0.914	−0.12 (−2.35∼2.11)
Echocardiographic examination				
LVEDD, mm	0.05	2.07	0.039	0.09 (0.01∼0.18)
LVESD, mm	0.04	1.23	0.218	0.05 (−0.03∼0.14)
IVSd, mm	0.13	−0.56	0.578	−0.07 (−0.33∼0.19)
LVPWd, mm	0.08	−0.42	0.672	−0.03 (−0.19∼0.12)
LA-ap, mm	0.04	−1.33	0.185	−0.05 (−0.12∼0.02)
AO-a, mm	0.02	0.38	0.703	0.01 (−0.03∼0.05)
AO-s, mm	0.05	1.43	0.153	0.07 (−0.03∼0.17)
AO-asc, mm	0.05	−1.90	0.059	−0.09 (−0.19∼0.00)
MPA, mm	0.09	−1.86	0.064	−0.17 (−0.34∼0.01)
EDV, ml	0.01	2.17	0.031	0.02 (0.01∼0.04)
ESV, ml	0.01	1.23	0.219	0.02 (−0.01∼0.05)
EF, %	0.01	0.22	0.825	0.00 (−0.01∼0.01)
FS, %	0.04	−0.53	0.593	−0.02 (−0.09∼0.05)
SV, ml	0.01	1.88	0.061	0.03 (−0.00∼0.05)
CO, ml/min	0.00	0.52	0.601	0.00 (−0.00∼0.00)
CI, ml/min/m²	0.00	−0.97	0.331	−0.00 (−0.00∼0.00)
MV E, m/s	1.02	−1.72	0.085	−1.77 (−3.77∼0.24)
MV A, m/s	0.91	−7.13	<.001	−6.48 (−8.26∼−4.70)
E/A	0.60	3.73	<.001	2.24 (1.06∼3.42)
AV, m/s	0.38	−0.20	0.841	−0.08 (−0.83∼0.67)
PV, m/s	0.78	−1.42	0.157	−1.10 (−2.63∼0.42)
e's, cm/s	0.10	4.14	<.001	0.40 (0.21∼0.59)
e'l, cm/s	0.07	3.76	<.001	0.28 (0.13∼0.43)
E/e	0.06	−5.36	<.001	−0.30 (−0.41∼−0.19)
Laboratory testing				
RHR, bpm	0.01	−9.77	<.001	−0.14 (−0.16∼−0.11)
RBC, 10^12^/L	0.33	10.12	<.001	3.30 (2.66∼3.94)
Hb, g/L	0.01	11.86	<.001	0.13 (0.11∼0.15)
HCT, %	0.04	8.28	<.001	0.32 (0.25∼0.40)
MCV, fL	0.03	−0.32	0.746	−0.01 (−0.06∼0.04)
TG, mmol/L	0.08	0.41	0.680	0.03 (−0.12∼0.19)
TC, mmol/L	0.12	1.30	0.193	0.16 (−0.08∼0.39)
LDL-C, mmol/L	0.08	1.19	0.236	0.10 (−0.06∼0.26)
HDL-C, mmol/L	0.02	2.31	0.021	0.05 (0.01∼0.10)
FBG, mmol/L	0.07	−1.59	0.112	−0.11 (−0.24∼0.03)
CK, U/L	0.00	2.19	0.029	0.01 (0.01∼0.01)

S.E, standard error; *t*, t-statistic; P, *P*-value; β, regression coefficient; CI, confidence interval.

### 3.3 Multivariable linear regression analysis

**Figure 2:**
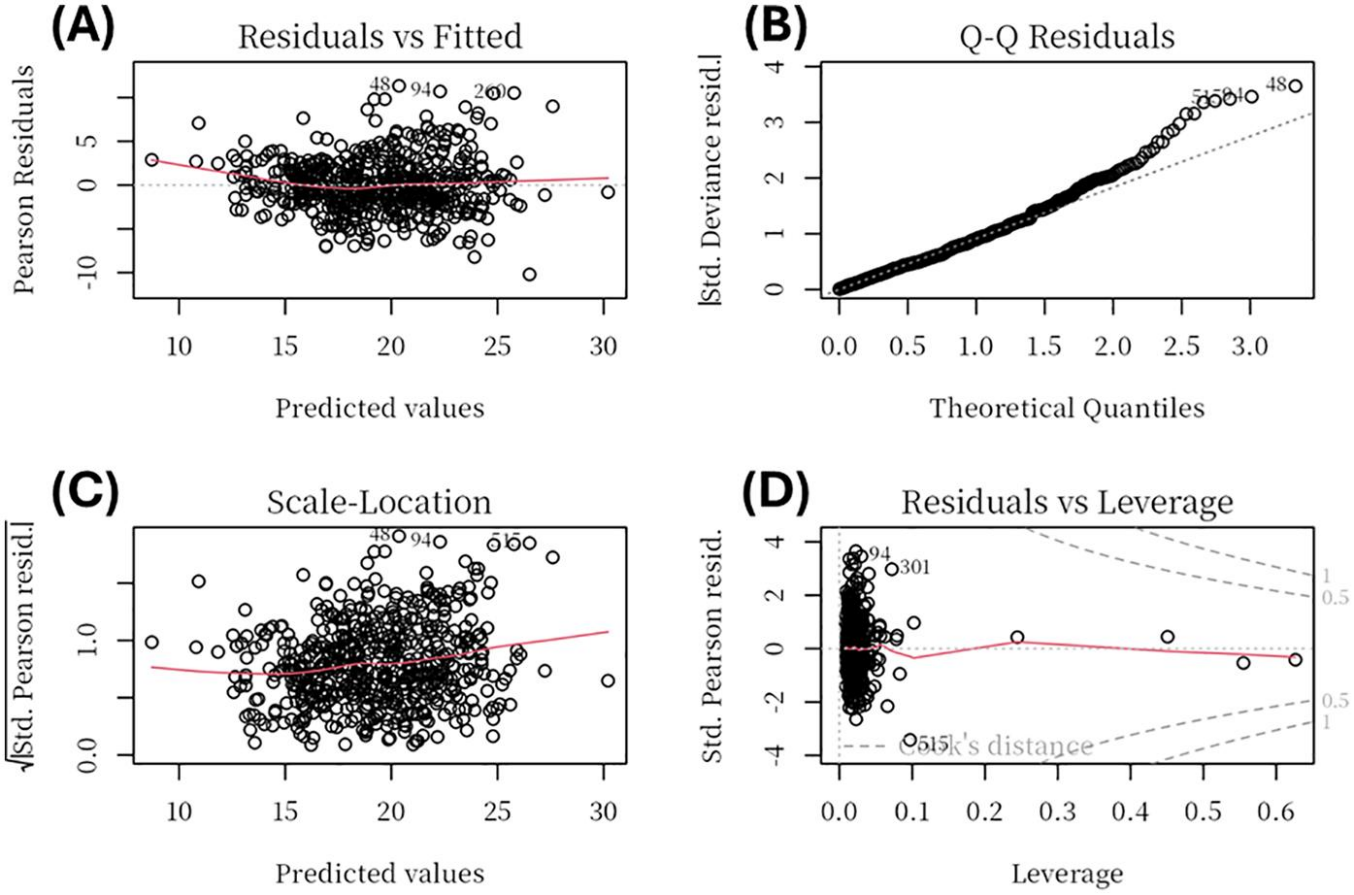
Regression diagnostic plots for the multivariable linear regression model. Diagnostic plots include: **(A)** Residuals vs Fitted, showing no major deviation from linearity; **(B)** Normal Q-Q plot, indicating that residuals were approximately normally distributed with minor deviations at the tails; **(C)** Scale–Location plot, suggesting homoscedasticity of residuals across fitted values; and **(D)** Residuals vs Leverage, used to identify potential high-leverage observations, none of which exerted undue influence.

To ensure robustness, we examined regression diagnostics. Multicollinearity was assessed using VIF. To ensure robustness, we examined regression diagnostics. Multicollinearity was assessed using VIF. Variables with excessive collinearity, particularly those reflecting overlapping echocardiographic constructs (LVEDD, VIF = 13.5; EDV, VIF = 13.4; MV A, VIF = 5.5; E/e, VIF = 7.0), were excluded during the stepwise selection process. The final model retained clinically relevant predictors, all of which demonstrated acceptable collinearity (VIF < 5; Supplementary Table S3). Model assumptions of linearity, homoscedasticity, and residual normality were further confirmed graphically (Figure 2).

Multivariable linear regression was conducted to identify independent predictors of VO₂peak (Table 3). Among demographic variables, Age (*β* = –0.06, *P* < 0.001), Female (*β* = –1.71, *P* < 0.001), ACS (*β* = –1.01, *P* < 0.001), Smoking (*β* = –2.37, *P* < 0.001), and have Hypertension (*β* = –0.82, *P* = 0.004) were significantly associated with lower VO₂peak. In addition, RHR (*β* = –0.10, *P* < 0.001) and HCT (*β* = –0.20, *P* = 0.002) were independently negatively associated, while RBC (*β* = 1.20, *P* = 0.012) and Hb (*β* = 0.09, *P* < 0.001) showed positive associations with VO₂peak.

**Table 3:** Multivariate regression of clinical parameters associated with peak VO₂.

Characteristics	S. E	t	P	β (95% CI)
Age, years	0.02	−3.97	<.001	−0.06 (−0.09∼−0.03)
Hight, cm	0.02	1.42	0.155	0.04 (−0.01∼0.08)
E/e	0.05	−1.58	0.114	−0.07 (−0.16∼0.02)
RHR, bpm	0.01	−8.07	<.001	−0.10 (−0.12∼−0.07)
RBC, 10^12^/L	0.47	2.53	0.012	1.20 (0.27∼2.13)
Hb, g/L	0.02	4.65	<.001	0.09 (0.05∼0.13)
HCT, %	0.06	−3.13	0.002	−0.20 (−0.32∼−0.07)
HDL-C, mmol/L	0.02	1.79	0.074	0.03 (−0.00∼0.06)
Gende				
Male				0.00 (Reference)
Female	0.45	−3.79	<.001	−1.71 (−2.59∼−0.82)
Type of CAD				
SA				0.00 (Reference)
ACS	0.29	−3.44	<.001	−1.01 (−1.59∼−0.44)
Smoking				
No				0.00 (Reference)
Yes	0.33	−7.14	<.001	−2.37 (−3.02∼−1.72)
Hypertension				
No				0.00 (Reference)
Yes	0.29	−2.86	0.004	−0.82 (−1.38∼−0.26)
Diabetes				
No				0.00 (Reference)
Yes	0.33	−1.61	0.108	−0.52 (−1.16∼0.11)

S.E, standard error; *t*, *t*-statistic; P, *P*-value; β, regression coefficient; CI, confidence interval.

### 3.4 Subgroup analysis

Subgroup analyses by age (cut-off at 65 years) are presented in Table 4. In both age groups, lower resting heart rate, higher hemoglobin, absence of ACS, and absence of smoking were independently associated with higher VO₂peak (all *P* < 0.05, 95% CI excluding 0). Distinct age-specific predictors were also observed. Among participants aged <65 years, lower MPA (*P* = 0.014, 95% CI: −5.47 to −0.64) and lower HCT (*P* = 0.022, 95% CI: −0.31 to −0.02) were unique independent predictors of reduced VO₂peak. In contrast, among those aged ≥65 years, hypertension (*P* < 0.001, 95% CI: −2.14 to −0.69) was a significant independent predictor. Although several echocardiographic parameters (LVEDD, EDV, SV, MV E, MV A, E/e) were significant in univariable analyses for the older group, they were excluded during the stepwise selection process due to a VIF > 5.

**Table 4:** Linear regression analyses of VO₂peak predictors by Age subgroup.

Characteristics	Aged <65 Years					Aged ≥65 Years				
	Univariable			Multivariable		Univariable			Multivariable	
	P	β (95% CI)		P	β (95%CI)	P	β (95% CI)		P	β (95% CI)
Hight, cm	<.001	0.14 (0.08∼0.21)				<.001	0.12 (0.06∼0.17)		0.104	0.05 (−0.01∼0.12)
LVEDD	-					0.004	0.17 (0.06∼0.28)		-	
MPA, mm	<.001	−5.49 (−8.40∼−2.58)		0.014	−3.06 (−5.47∼−0.64)	-				
EDV	-					0.003	0.03 (0.01∼0.05)		-	
SV	-					0.003	0.05 (0.02∼0.09)			
MV E	-					0.036	−2.93 (−5.65∼−0.21)		-	
MV A	-					<.001	−4.01 (−6.28∼−1.74)			
E/e	-					<.001	−0.22 (−0.35∼−0.09)		-	
E's, cm/s	0.010	0.37 (0.09∼0.66)		-		-				
RHR, bpm	<.001	−0.14 (−0.19∼−0.10)		<.001	−0.10 (−0.14∼−0.06)	<.001	-−0.12 (−0.15∼−0.08)		<.001	−0.08 (−0.11∼−0.06)
RBC, 10^12^/L	<.001	2.81 (1.86∼3.76)		-		<.001	2.83 (1.97∼3.69)		0.070	1.10 (−0.08∼2.29)
Hb, g/L	<.001	0.12 (0.09∼0.15)		<.001	0.11 (0.07∼0.16)	<.001	0.11 (0.08∼0.13)		0.026	0.05 (0.01∼0.09)
HCT, %	<.001	0.23 (0.12∼0.33)		0.022	−0.17 (−0.31∼−0.02)	<.001	0.34 (0.24∼0.44)		-	
Gender										
Male		0.00 (Reference)			0.00 (Reference)		0.00 (Reference)			0.00 (Reference)
Female	<.001	−3.05 (−4.10∼−2.00)		<.001	−2.33 (−3.45∼−1.21)	<.001	-−2.55 (−3.46∼−1.65)		0.011	−1.51 (−2.66∼−0.35)
Type of CAD										
SA		0.00 (Reference)			0.00 (Reference)		0.00 (Reference)			0.00 (Reference)
ACS	0.010	−1.44 (−2.53∼−0.36)		0.005	−1.30 (−2.20∼−0.41)	0.030	-−0.99 (−1.88∼−0.10)		0.018	−0.86 (−1.56∼−0.15)
Smoking										
No		0.00 (Reference)			0.00 (Reference)		0.00 (Reference)			0.00 (Reference)
Yes	<.001	−2.25 (−3.43∼−1.07)		<.001	−2.81 (−3.85∼−1.77)	0.019	-−1.09 (−1.99∼−0.18)		<.001	−2.30 (−3.10∼−1.49)
Hypertension				-						
No		0.00 (Reference)					0.00 (Reference)			0.00 (Reference)
Yes	0.016	−1.27 (−2.30∼−0.24)				<.001	−2.16 (−3.05∼−1.26)	<.001	−1.42 (−2.14∼−0.69)	
Diabetes	-							--		
No	-						0.00 (Reference)			
Yes	-					0.018	-−1.22 (−2.22∼−0.22)			

P, *P*-value; β, regression coefficient; CI, confidence interval. Univariable = univariable linear regression analysis; Multivariable = multivariable linear regression analysis. “–” indicates non-significant results (*P* > 0.05) in either univariable or multivariable models.

Subgroup analyses by gender are presented in Table 5. In both males and females, lower resting heart rate, higher hemoglobin, absence of ACS, and absence of smoking were independently associated with higher VO₂peak (all *P* < 0.05, 95% CI excluding 0). Additionally, older age remained an independent predictor of lower VO₂peak in both genders (male: *P* < 0.001, 95% CI: −0.14 to −0.07; female: *P* = 0.003, 95% CI: −0.11 to −0.02). However, there were gender-specific associations. Among males, hypertension (*P* = 0.012, 95% CI: −1.60 to −0.20) and higher HDL-C (*P* = 0.047, 95% CI: 0.01–0.07) were independent predictors of VO₂peak, while these associations were not observed in females. Conversely, in females, lower body weight (*P* = 0.008, 95% CI: −0.12 to −0.02), higher RBC (*P* < 0.001, 95% CI: 1.50–4.58), and lower HCT (*P* = 0.006, 95% CI: −0.84 to −0.15) were independently associated with VO₂peak, which were not significant in males.

**Table 5:** Linear regression analyses of VO₂peak predictors by gender.

Characteristics	Male					Female				
	Univariable			Multivariable		Univariable			Multivariable	
	P	β (95% CI)		P	β (95% CI)	P	β (95%CI)		P	β (95% CI)
Age, years	<.001	−0.16 (−0.20∼−0.12)		<.001	−0.11 (−0.14∼−0.07)	<.001	−0.12 (−0.16∼−0.07)		0.003	−0.07 (−0.11∼−0.02)
Weight, kg	-					0.008	−0.07 (−0.12∼−0.02)		<.001	−0.10 (−0.14∼−0.06)
IVSd	-					0.004	−0.57 (−0.95∼−0.18)		-	
LA-ap	-					0.012	−0.15 (−0.27∼−0.03)		-	
AO-asc	0.003	−0.19 (−0.31∼−0.06)				-				
MPA	-					0.030	−0.29 (−0.54∼−0.03)		-	
MV A	<.001	−6.80 (−9.24∼−4.37)	-			<.001	−3.82 (−6.01∼−1.62)		-	
E/A	<.001	3.17 (1.59∼4.75)	-			-				
E's	<.001	0.43 (0.19∼0.67)	-			-				
E'l	<.001	0.33 (0.14∼0.53)	-			-				
E/e	<.001	−0.32 (−0.49∼−0.14)	-			0.008	−0.16 (−0.27∼−0.04)		0.081	−0.09 (−0.20∼0.01)
RHR, bpm	<.001	−0.16 (−0.19∼−0.12)		<.001	−0.12 (−0.15∼−0.09)	<.001	−0.07 (−0.10∼−0.04)		<.001	−0.07 (−0.10∼−0.04)
RBC, 10^12^/L	<.001	2.90 (2.05∼3.76)	-			<.001	2.32 (1.29∼3.34)		<.001	3.04 (1.50∼4.58)
Hb, g/L	<.001	0.13 (0.10∼0.16)		<.001	0.10 (0.06∼0.14)	<.001	0.08 (0.04∼0.11)		0.006	0.12 (0.03∼0.20)
HCT, %	<.001	0.25 (0.15∼0.35)		0.161	−0.09 (−0.21∼0.04)	0.002	0.22 (0.08∼0.35)		0.006	−0.49 (−0.84∼−0.15)
TG, mmol/L	0.014	0.38 (0.08∼0.67)		0.161	0.16 (−0.07∼0.39)	-				
HDL-C, mmol/L	0.049	0.05 (0.01∼0.09)		0.047	0.03 (0.01∼0.07)	-				
Type of CAD										
SA		0.00 (Reference)			0.00 (Reference)		0.00 (Reference)			0.00 (Reference)
ACS	<.001	−1.93 (−2.83∼−1.03)		0.014	−0.90 (−1.62∼−0.19)	0.018	−1.24 (−2.26∼−0.22)		0.131	−0.66 (−1.52∼0.19)
Smoking										
No		0.00 (Reference)			0.00 (Reference)		0.00 (Reference)			0.00 (Reference)
Yes	<.001	−3.81 (−4.64∼−2.98)		<.001	−2.16 (−2.87∼−1.44)	0.005	−3.10 (−5.23∼−0.97)		0.007	−2.40 (−4.13∼−0.68)
Hypertension						-				
No		0.00 (Reference)			0.00 (Reference)	-				
Yes	<.001	−2.15 (−3.03∼−1.28)		0.012	−0.90 (−1.60∼−0.20)	-				
Diabetes				-		-				
No		0.00 (Reference)		-		-				
Yes	0.002	−1.64 (−2.69∼−0.60)		-		-				

P, *P*-value; β, regression coefficient; CI, confidence interval. Univariable = univariable linear regression analysis; Multivariable = multivariable linear regression analysis. “–” indicates non-significant results (*P* > 0.05) in either univariable or multivariable models.

### 3.5 Sensitivity analyses

Compared with included patients (*n* = 575), those excluded (*n* = 462) due to incomplete or subquality CPET were significantly older and heavier, and had higher proportions of ACS, current smoking, hypertension, and hyperlipidemia. There were no significant differences in sex distribution, diabetes, height, or BMI categories. CPET indices and laboratory tests were largely unavailable for the excluded participants and were not compared. Details shown in Supplementary Table S4.

## 4 Discussion

In the present study, we assessed cardiopulmonary exercise capacity (VO₂peak) and its independent predictors among 575 post-PCI patients with CAD from Fujian, China. At baseline, participants in the lowest VO₂peak quartile were older, had higher resting heart rates, and lower hemoglobin and hematocrit levels. They also had higher rates of hypertension, diabetes, and ACS, while males and stable CAD were more common in the higher quartiles. Significant differences in echocardiographic and laboratory parameters were also observed between groups. Multivariable analysis showed that lower VO₂peak was independently associated with older age, female sex, ACS, current smoking, hypertension, and a higher RHR. Conversely, RBC count and Hb were positively associated with VO₂peak, whereas lower HCT was negatively associated. Subgroup analyses revealed distinct age- and sex-specific determinants of VO₂peak. In participants aged <65 years, a larger MPA and lower HCT independently predicted reduced VO₂peak, whereas these associations were absent in those ≥65 years. Hypertension independently predicted lower VO₂peak only in participants aged ≥65 years. This may be explained by age-related physiological changes, including increased vascular stiffness and impaired endothelial function, which elevate afterload and reduce diastolic reserve (36, 37). These changes make older adults more susceptible to the detrimental effects of hypertension on stroke volume and overall exercise capacity. In contrast, younger patients (<65 years) typically retain better vascular compliance and diastolic function, which likely mitigates the impact of hypertension on VO₂peak. Notably, only MPA remained significant among echocardiographic indices in younger participants, likely due to multicollinearity among LV size/function measures (e.g., EF with EDV/ESV; SV–CO–CI; E/e′ with E and e′), which reduces unique explanatory variance after mutual adjustment (despite VIF < 5). By contrast, MPA captures a relatively orthogonal cardiopulmonary load (RV–pulmonary vascular coupling), providing independent information relevant to exercise limitation in younger patients. Sex-stratified models showed that hypertension and higher HDL-C were independent predictors in males, while lower body weight, higher RBC count, and lower HCT were uniquely associated with VO₂peak in females. These findings suggest that hematologic factors, particularly RBC count and HCT, may have a stronger impact on oxygen delivery and cardiopulmonary fitness in females compared to males. In females patients, these factors may interact with sex-specific physiological mechanisms such as hormonal influences and iron metabolism (38). These findings suggest that monitoring and managing hematologic health in female patients may have potential benefits for cardiopulmonary fitness (39). These divergences underscore the need for tailored rehabilitation programmes that take age and sex into account.

Our study found that the average VO₂peak in post-PCI CAD patients was 19.29 ± 4.41 ml/kg/min (males: 20.21 ± 4.54 ml/kg/min, females: 17.37 ± 3.41 ml/kg/min), which aligns with previous global reports. For example, Rasch-Halvorsen et al. observed VO₂peak values of 23.8 ± 6.4 ml/kg/min in men and 19.7 ± 4.4 ml/kg/min in women in a Norwegian study (40). Similarly, a U.S. study reported VO₂peak values of 15.2 ± 4.0 ml/kg/min in females and 21.2 ± 7.1 ml/kg/min in males with PCI without MI (41). In contrast, a study from Shanghai, China, reported slightly lower values (17.54 ± 3.38 ml/kg/min) (42), while a study from Hefei, China, found a VO₂peak of 19.9 ± 4.5 ml/kg/min in post-PCI patients (43). These variations in VO₂peak can be attributed to factors such as population demographics, healthcare access, rehabilitation strategies, and lifestyle differences. Our analysis further confirmed that older age, female sex, smoking, hypertension, ACS, and higher resting heart rate were independently associated with lower cardiopulmonary exercise capacity in post-PCI CAD patients (44–49).

Large cohort studies, such as the Baltimore Longitudinal Study of Aging (50), have shown a progressive decline in VO₂peak with advancing age, accelerating markedly after age 70, and with men experiencing a more rapid reduction after the age of 40. These trends likely reflect both age-related vascular and metabolic decline, as well as underlying cardiovascular pathology. Sex differences in cardiopulmonary exercise capacity are primarily attributable to higher cardiac output, blood volume, and hemoglobin levels in men, while hormonal fluctuations, particularly in estrogen, further influence exercise capacity trajectories in women (51, 52). These factors together contribute to generally higher VO₂peak values in males compared to females. Additionally, smokers with CAD exhibit more severe atherosclerosis and myocardial dysfunction, resulting in lower VO₂peak and increased cardiovascular risk (53, 54). Comprehensive rehabilitation in these patients should integrate smoking cessation with individualized exercise interventions. Post-PCI patients and concomitant hypertension typically have lower VO₂peak than those without hypertension, mainly because chronic hypertension leads to increased arterial stiffness, impaired vascular function, and cardiac remodelling, all of which limit cardiopulmonary exercise capacity (55–57). Patients with ACS demonstrate significantly lower VO₂peak compared to those with stable CAD, likely due to greater myocardial injury and heightened systemic inflammation. These pathological differences also contribute to a higher risk of recurrent ischemic events in ACS patients (58–60). These findings emphasize the need for individualized rehabilitation strategies tailored to high-risk subgroups to optimize cardiopulmonary recovery following PCI. RHR also emerged as an independent negative predictor of VO₂peak, reflecting impaired cardiovascular adaptation. This finding is consistent with results from Kato et al., who analyzed a single-center data of 2,160 Asian cardiovascular patients undergoing CPET and demonstrated a significant negative association between RHR and VO₂peak (Ptrend < 0.0001) (61). Evidence further suggests that rehabilitation interventions targeting reductions in RHR can improve exercise capacity in coronary heart disease patients (62, 63).

Beyond traditional risk factors, our findings emphasize the importance of hematologic parameters in predicting VO₂peak. Lower levels of Hb were correlated with decreased cardiopulmonary exercise capacity, consistent with prior research in Asian populations (64). Previous studies have also demonstrated that postoperative anemia due to low Hb is an independent predictor of increased mortality and is associated with higher incidence of major adverse cardiac events, increased troponin levels, elevated creatine kinase-MB fractions, and prolonged hospital stays within 30 days post-PCI (65). Therefore, monitoring Hb levels closely after PCI and implementing personalized aerobic rehabilitation programs, such as low-intensity endurance training, are crucial for optimizing long-term recovery and cardiopulmonary fitness. Additionally, our results indicate that lower RBC counts were associated with reduced cardiopulmonary exercise capacity in post-PCI CAD patients. RBC count is well established as an important marker of cardiac function and cardiovascular health (66, 67). A recent study reported significant reductions in cardiovascular mortality risk with incremental increases in RBC counts in heart failure patients, with a 28% decrease observed in males and 43% in females for every increment of 1.0 × 10¹²/L (68). Thus, incorporating RBC count monitoring into cardiovascular health assessments post-PCI, particularly for patients with heart failure, or diabetes, may significantly enhance cardiopulmonary recovery by improving oxygen delivery capacity. Although diabetes was associated with lower VO₂peak in univariable analyses, the effect was attenuated to non-significance after adjustment. This pattern is consistent with confounding/partial mediation by correlated factors—particularly hypertension and lower hemoglobin/hematocrit, as well as adiposity and dyslipidemia—that more proximally influence exercise capacity.

Clinically, our findings underscore the importance of comprehensive assessments—including cardiac function and biochemical screening—in developing precise rehabilitation and secondary prevention strategies post-PCI. The distinct age- and sex-specific determinants identified in our subgroup analyses suggest that younger patients (<65 years) with reduced HCT or larger MPA may benefit from targeted strategies aimed at improving oxygen-carrying capacity and monitoring cardiac structural adaptations. Conversely, older patients (≥65 years) may require more intensive management of hypertension to enhance cardiopulmonary fitness. Sex-specific rehabilitation programs should focus on blood pressure and lipid management for males, while interventions aimed at improving oxygen delivery. Maintaining optimal body weight and addressing low HCT may be particularly beneficial for females. In this group, lower HCT levels were correlated with reduced oxygen-carrying capacity and VO₂peak. Interventions such as iron supplementation or dietary adjustments to support red blood cell production may help improve oxygen delivery during exertion, potentially contributing to better functional outcomes in women post-PCI, particularly in those with lower HCT levels. These tailored approaches enable clinicians to personalize exercise prescriptions effectively and optimize long-term outcomes in CAD patients following PCI.

Our study has several limitations. First, its cross-sectional design restricts causal inference, and as a single-center study, the exclusion of 462 patients due to suboptimal CPET quality may not be generalizable to broader populations. Second, although all participants underwent CPET within 6 weeks after PCI, the exact timing varied across individuals, which could introduce residual confounding because cardiopulmonary exercise capacity may improve during early recovery. To minimize this concern, we excluded patients with complications that could markedly affect short-term exercise capacity and additionally performed a sensitivity analysis restricting CPET timing to a narrower window, which yielded consistent results. Third, sufficient baseline pre-PCI exercise capacity data were lacking, limiting our ability to differentiate post-PCI changes from preexisting interindividual differences. Fourth, relevant factors such as habitual physical activity levels and psychological status were not assessed. Future studies should incorporate long-term follow-up to monitor recurrent events and functional outcomes and expand to multicentre cohorts to enhance representativeness and robustness.

## 5 Conclusions

This study identified key demographic, clinical, echocardiographic, and biochemical predictors of cardiopulmonary exercise capacity (VO₂peak) in post-PCI patients with coronary artery disease in Fujian, China. Importantly, age- and sex-specific differences in these associations were observed, highlighting the need for individualized rehabilitation and secondary prevention strategies. These findings provide a foundation for precision cardiopulmonary management to improve long-term outcomes in this high-risk population.

## Data Availability

The raw data supporting the conclusions of this article will be made available by the authors, without undue reservation.
