# Supplementary material for: Association between cardiopulmonary exercise capacity and clinical parameters in post-PCI patients with coronary artery disease from Fujian, China
Source: Front Cardiovasc Med. 2025 Sep 25;12:1674787. doi: 10.3389/fcvm.2025.1674787 (PMC12507881; doi:10.3389/fcvm.2025.1674787)
Supplement: Supplementary file 2 [file Datasheet2.pdf]

Methods supplement

Standardized Cardiopulmonary Exercise Testing (CPET) Protocol

This study followed the *Chinese expert consensus on standardized clinical application of cardiopulmonary exercise testing* (2022) and international practice guidelines. The CPET protocol was standardized as follows.

1. Contraindications for CPET

Contraindications were screened prior to testing, in accordance with the expert consensus

Absolute contraindications included uncontrolled acute coronary syndromes, acute heart failure, severe aortic stenosis or regurgitation, acute myocarditis or pericarditis, uncontrolled arrhythmias, acute pulmonary embolism, severe hypoxemia (resting SpO_2 <85%), acute deep venous thrombosis, or lack of informed consent.

Relative contraindications included known coronary stenosis ⩾50% without revascularization, moderate valvular disease, hypertrophic cardiomyopathy, severe pulmonary hypertension, uncontrolled hypertension (SBP ⩾180 mmHg or DBP ⩾110 mmHg), resting HR ⩾120 bpm, untreated endocrine or hematologic disorders (e.g., thyroid dysfunction, severe anemia, electrolyte imbalance), musculoskeletal injury, or mental disorders limiting exercise cooperation.

2. Pre-test Preparation

Environment: The CPET laboratory area was ⩾20 m^2, equipped with ergometers, monitoring systems, and resuscitation equipment (defibrillator, oxygen supply, emergency medications). Room temperature was maintained at 20–22 °C, humidity at ~50%, with adequate lighting, ventilation, and privacy. A Borg rating of perceived exertion (RPE) scale was displayed on the wall.

Calibration: Gas analyzers and flow sensors were calibrated daily. O_2 and CO_2 analyzers were calibrated with reference gases before each test.

Participant preparation: Patients were required to be clinically stable, wear comfortable clothing and footwear, and fast for at least 2 h before testing. Caffeine, alcohol, and smoking were avoided within 2 h of testing. Patients were fitted with a face mask (or mouthpiece + nose clip) and instructed in the use of the Borg RPE scale.

Personnel preparation: Tests were supervised by trained physicians and technicians with certification in advanced life support.

3. Test Procedure

1. Application and physician evaluation: Each patient was assessed for medical history, current medication, smoking, daily activity level, and contraindications. Informed consent was obtained.

2. Resting period: A 3-minute seated rest with ECG, blood pressure, and respiratory parameters recorded.

3. Warm-up: A 3-minute unloaded pedaling at 0 W with cadence 55–65 rpm.

4. Ramp protocol: Workload was increased continuously at a rate of 10–30 W/min, individualized by the supervising physician according to health status, physical fitness, and anticipated exercise tolerance. Ramp rate selection followed consensus recommendations, aiming for volitional exhaustion in ~10–15 minutes. Frail patients were typically assigned 10–15 W/min increments, while fitter patients were assigned 20–30 W/min.

Formulae (per consensus):

- Predicted unloaded VO_2 (ml/min) = 150 + (6 × body weight [kg])
- Predicted peak VO_2 (ml/min) = (height [cm] − age [y]) × 20 (male) or ×14 (female)
- Recommended ramp rate (W/min) = (Predicted peak VO_2 − Predicted unloaded VO_2) / 100

5. Monitoring: ECG, blood pressure, oxygen saturation, respiratory gases, and patient symptoms were continuously recorded.

6. Recovery phase: After termination, patients pedaled unloaded for 2–3 minutes at 30–40 rpm, then were observed for 6–8 minutes until vital signs returned near baseline.

4. Test Termination Criteria

The test was terminated if:

- Patient reached 75–85% of age-predicted maximal HR or RER >1.05;
- Development of moderate to severe angina, severe dyspnea, dizziness, ataxia, pallor, or cyanosis;
- ST-segment depression ⩾0.20 mV for ⩾2 min or elevation ⩾0.10 mV with arched upward configuration;
- Severe arrhythmias (ventricular tachycardia, frequent PVCs, AF with rapid ventricular response, AV block ⩾ second degree);
- SBP drop ⩾10 mmHg with increasing workload or SBP >220 mmHg / DBP >110 mmHg;
- Musculoskeletal fatigue or cramps leading to cadence decline;

Patient request to stop.

5. Safety

All tests were performed under direct supervision of an experienced cardiologist, with emergency equipment available.
